# Continuous Carryover of Temporal Context Dissociates Response Bias from Perceptual Influence for Duration

**DOI:** 10.1371/journal.pone.0100803

**Published:** 2014-06-25

**Authors:** Martin Wiener, James C. Thompson, H. Branch Coslett

**Affiliations:** 1 Department of Neurology, University of Pennsylvania, Philadelphia, Pennsylvania, United States of America; 2 Department of Psychology, George Mason University, Fairfax, Virginia, United States of America; Duke University, United States of America

## Abstract

Recent experimental evidence suggests that the perception of temporal intervals is influenced by the temporal context in which they are presented. A longstanding example is the time-order-error, wherein the perception of two intervals relative to one another is influenced by the order in which they are presented. Here, we test whether the perception of temporal intervals in an absolute judgment task is influenced by the preceding temporal context. Human subjects participated in a temporal bisection task with no anchor durations (partition method). Intervals were demarcated by a Gaussian blob (visual condition) or burst of white noise (auditory condition) that persisted for one of seven logarithmically spaced sub-second intervals. Crucially, the order in which stimuli were presented was first-order counterbalanced, allowing us to measure the carryover effect of every successive combination of intervals. The results demonstrated a number of distinct findings. First, the perception of each interval was biased by the prior response, such that each interval was judged similarly to the preceding trial. Second, the perception of each interval was also influenced by the prior interval, such that perceived duration shifted away from the preceding interval. Additionally, the effect of decision bias was larger for visual intervals, whereas auditory intervals engendered greater perceptual carryover. We quantified these effects by designing a biologically-inspired computational model that measures noisy representations of time against an adaptive memory prior while simultaneously accounting for uncertainty, consistent with a Bayesian heuristic. We found that our model could account for all of the effects observed in human data. Additionally, our model could only accommodate both carryover effects when uncertainty and memory were calculated separately, suggesting separate neural representations for each. These findings demonstrate that time is susceptible to similar carryover effects as other basic stimulus attributes, and that the brain rapidly adapts to temporal context.

## Introduction

The processing of any stimulus feature requires an understanding of the temporal context in which it occurs. For example, in order to properly process music or speech, the brain must learn to predict and adapt to rapidly changing stimuli. The ability to learn temporal relationships depends on our ability to perceive differences in duration. However, the processing of duration remains an elusive aspect of psychology and neuroscience [1], [2].

Recent research has suggested that the brain adapts to changes in duration in a manner similar to other stimulus features, such as orientation and size, such that repeated presentations of the same duration lead to a contraction of perceived time that is relative to the adapter duration [3]. Separately, researchers have also shown that timed responses exhibit central tendency [4], where response times trend towards the mean of previously experienced stimuli [5], [6]. Here, we present data and computational modeling suggesting that both effects are manifestations of the same underlying process. More specifically, we provide evidence that the brain continuously adapts to temporal context.

A core feature of duration processing is that temporal estimates are noisy. Noisy estimations entail a level of uncertainty that scales with duration length, in accordance with Weber-Fechner psychophysical laws. Attempts to model this effect have utilized a variety of procedures, including pacemaker accumulation [7], perceptron-like processes [8], state-dependent readout [9], and most recently drift-diffusion processes [10]. However, the influence of the prior temporal context is generally ignored in these models, with the perception of each interval being independent of every other interval. Yet, between-trial changes, also known as carryover effects, may reveal hidden sources of variance and bias that can inform how the underlying representations were generated and decisional computations accomplished [11], [12].

Carryover effects can be broadly divided into two categories. *Perceptual* carryover refers to the influence of the preceding stimulus on the present stimulus, whereas *decisional* carryover is the influence of the preceding decision on the present decision [13], [14]. Both types of carryover can be either assimilative or contrastive; assimilative effects may relate to uncertainty in decision-making criteria, whereas contrastive effects may represent sensory adaptation. To explore both effects, we utilized a variant of the commonly used temporal bisection task, in which participants are presented with stimuli at a range of possible durations and must classify each stimulus into “short” or “long” duration categories [15]. In this variant, known as the partition method, no explicit anchors are provided, and so the categorical boundary for duration categories must be computed based on the statistics of presented stimuli. In this way, our task is more similar to absolute identification tasks than relative discrimination tasks, where subjects are explicitly comparing two quantities. Previous work has demonstrated that the bisection point, a measure of the categorical boundary between short and long, is typically located at the geometric mean of the stimulus set [16], and does not depend on the presence of anchors.

In our study, we sought to measure the carryover effect of both the previous stimulus duration and the previous decision on the present response. Previous work using absolute identification of time intervals has demonstrated that consecutive trial responses are positively correlated [17]; however, it is unclear whether this assimilation is caused by a change in perception, or a bias to respond similarly to the previous trial. To surmount this issue, the presentation order in our experiment was determined by a first-order counterbalanced de Bruijn sequence, in which every possible transition between each duration in our stimulus set occurred an equal number of times (Materials and Methods). Notably, any randomized sequence will become first-order counterbalanced with sufficient time; the advantage of the de Bruijn in our case was to minimize the number of trials necessary for complete counterbalancing, reducing the time for a single session to 15 minutes. By using a first-order counterbalanced sequence, we could separately measure the influence of every prior duration on the perception of every current duration. Furthermore, we could also measure the influence of every prior decision on every current response. We found that the perception of time is susceptible to similar adaptive and decisional effects as other categorical stimuli, where the responses for any given interval are simultaneously assimilated by the prior response and contrasted away from the prior interval. We quantified this effect by implementing a computational model of an ideal observer that incorporates an implicit memory prior distribution [18], which also estimates the uncertainty associated with any given estimate of time. These findings reveal a number of previously unknown phenomena for time perception, and provide further insight into the underlying computations necessary for adapting to changing temporal contexts.

## Results

**Figure 1:**
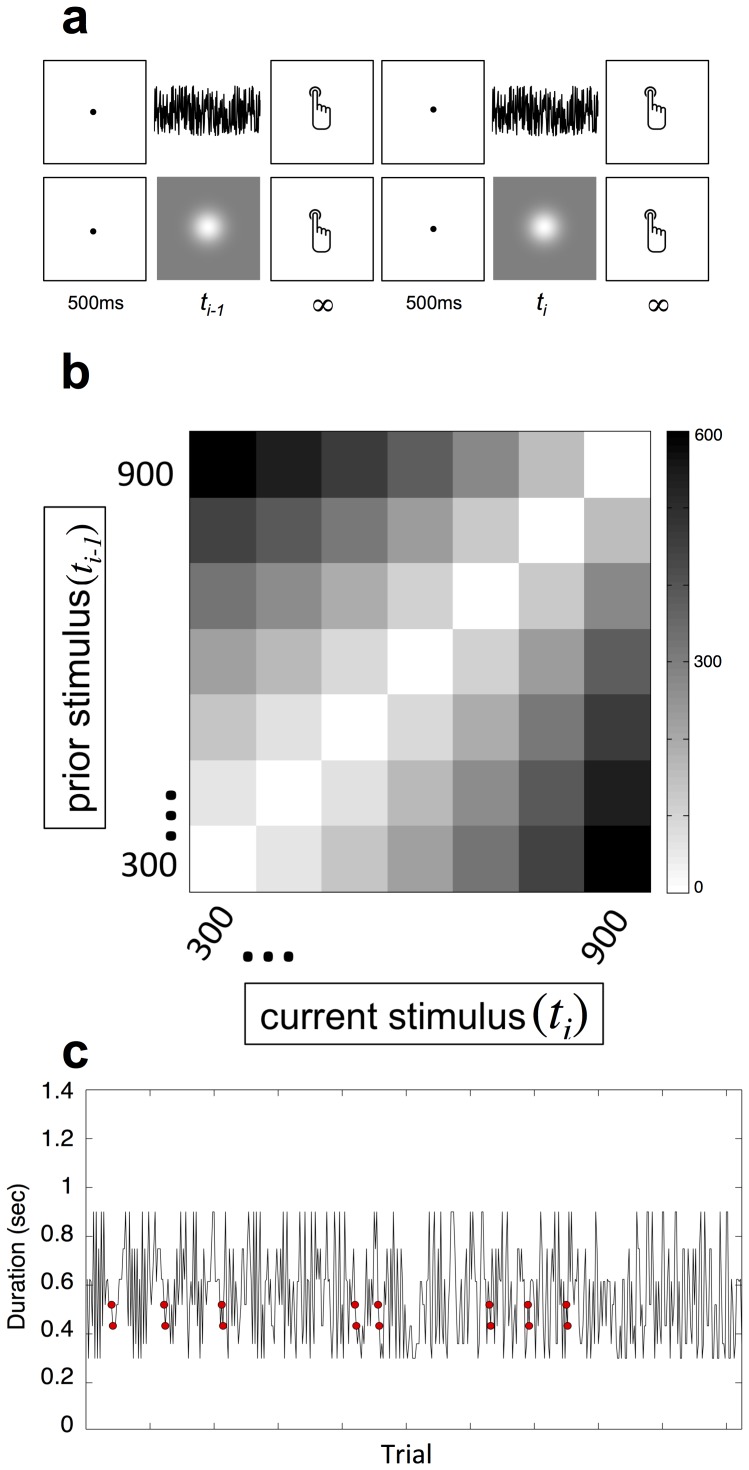
Task Design (a) Participants performed a 2AFC temporal bisection task, in which they were required to categorize whether an interval belonged to “short” or “long” categories. On a given trial, participants viewed a fixation point, followed by either a burst of white noise or a Gaussian blur, which persisted for one of seven durations. Participants were then required to respond as quickly but as accurately as possible which category the stimulus belonged to, which initiated the following trial. Separate groups participated in the auditory and visual versions of the task. (b) Temporal distance matrix for de Bruijn sequence. The stimulus set included seven logarithmically-spaced intervals between 300 and 900 ms. The presented matrix displays the distance, in ms, between every possible successive trial combination between current and prior durations. Direct effects are the influence of the present stimulus (*t_i_*) on a response whereas carryover effects are the influence of the preceding trial stimulus (*t_i_*_-1_). (c) Trial order determined by the path guided de Bruijn sequence was modulated by a random pairing of sinusoids, providing a perceptually stochastic sequence with relatively equal spacing of stimulus pairs throughout the entire session; red circles indicate a particular pair of trials (520–433 ms).

Human participants (n = 80) were tested on a speeded 2AFC temporal bisection task (see Methods and Figure 1a). Separate groups (n = 40 ea.) performed the bisection task with unimodal auditory or visual stimuli.

### Direct effects

**Figure 2:**
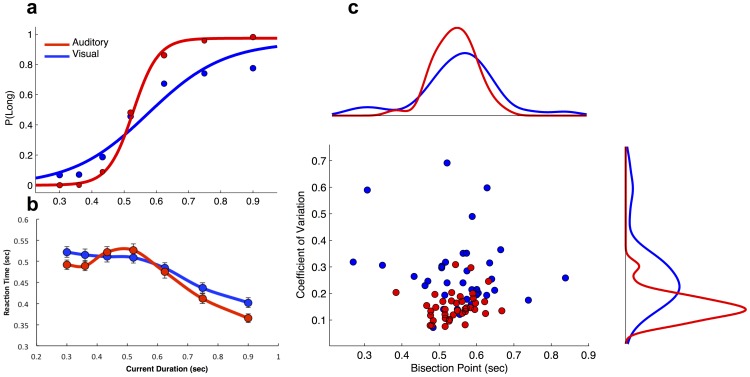
Direct effects of duration. (**a**) Temporal bisection performance for two representative subjects on different modality versions of the task. The probability of categorizing each duration as Long as well as the best fitting logistic function are displayed. (**b**) Average chronometric functions for auditory and visual participants. Reaction time decreased as a function of duration, but dropped off faster after the BP was passed and was faster for auditory stimuli at the longest duration tested. Error bars indicate s.e. of the mean. (**c**) Individual participant performance for the bisection point (x-axis) and coefficient of variation (y-axis) values derived from psychometric data, as well as marginal histograms for each; there were no differences between BP values for auditory or visual participants, but significantly lower CV values for the auditory task.

The de Bruijn sequence allowed us to divide our analysis into the direct and carryover effect of each stimulus (Figure 1b). For the direct effects, we replicated a number of well-known findings within the time perception literature. First, the bisection point (BP), a measure of the perceptual midpoint, occurred between the geometric and arithmetic means of the dataset for auditory and visual stimuli (Figure 2a,c), consistent with duration-spacing effects previously reported [16]. Second, the coefficient of variation (CV), a normalized index of perceptual variability (equivalent to the Weber Fraction), was significantly smaller for auditory than visual stimuli [*t*(70) = -17.985, *p*<0.0001] (Figure 2c), similar to previous findings that auditory stimuli are more precisely timed than visual stimuli [6]. Chronometric functions, derived from the reaction time (RT) for each stimulus, revealed that RT parametrically decreased as duration increased (Figure 2b), with faster RTs occurring after the BP had elapsed, consistent with response choice preparation findings [19].

### Carryover Effects

**Figure 3:**
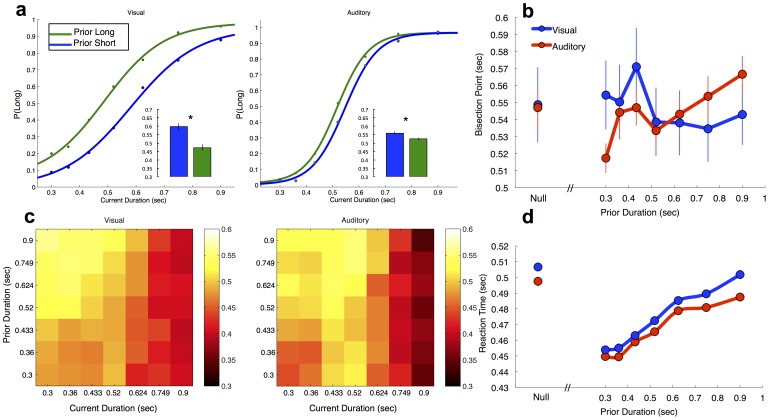
Carryover effects of duration. (**a**) Grand average psychometric curves for auditory and visual participants displaying the response for each duration on trials preceded by a response choice Long or Short. Insets display the average BP across participants for each prior choice condition; participants were more likely to classify an interval as long (or short) if the prior decision, independent of the prior interval, was classified as long (or short). Error bars indicate s.e. of the mean. Asterisks indicate significance at *p*<0.05. (**b**) Perceptual influence of the prior interval for visual and auditory participants. The far left point on each graph indicates the BP for the prior null condition; notably, the BP for the null prior condition was in the middle of the range of BPs observed for all prior duration conditions for both auditory and visual stimuli. Auditory participants exhibited a linear effect of prior interval, such that shorter prior intervals were associated with more leftward psychometric curves and smaller BPs, indicating a greater probability of categorizing stimuli as longer. No effect of prior interval was found for visual participants. (**c**) Response matrices displaying reaction time (RT) for every condition pairing. Slower RTs (hotter pixel colors) were found for shorter durations that were preceded by trials with longer durations. (**d**) RT data for each prior duration, averaged across current duration; both auditory and visual participants exhibited a strong linear effect of the prior duration.

For decisional carryover, we observed that the preceding decision had a strong impact on subsequent decisions (Figure 3a), such that responses were assimilated by the prior response [20]. The BP for stimuli on which the prior stimulus was judged as long was thus shifted leftward for both visual [*t*(35) = 4.717, *p*<0.0001] and auditory [*t*(38) = 6.852, *p*<0.0001] stimuli. This effect was significantly larger for visual stimuli [*F*(1,75) = 21.727, *p*<0.0001]; Additionally, there was no effect of prior decision on variability for auditory stimuli [*t*(38) = −0.713, *p* = 0.480], yet for visual stimuli the CV was significantly larger when the prior response choice was “long”, rather than “short” [*t*(35) = −2.149, *p* = 0.038].

For perceptual carryover, the effect of prior stimulus duration exhibited a contrastive effect on the perception of the present duration (Figure 3b). Specifically, the longer the prior interval, the more likely the following interval would be judged as short, and vice versa. This effect manifested as a linear effect on the BP; however, this was only exhibited for auditory [*F*(1,38) = 10.517, *p* = 0.002] and not visual [*F*(1,32) = 1.505, *p* = 0.229] stimuli. No effect of prior duration was observed on variability measures for either modality [Visual: *F*(1,32) = 0.383, *p* = 0.540; Auditory: *F*(1,38) = 0.493, *p* = 0.487].

For RT, we also noted a linear effect of prior duration on the present RT (Figure 3c,d), with shorter prior durations leading to faster reaction times. This effect was found for both auditory [*F*(1,38) = 76.591, *p*<0.0001] and visual [*F* (1,32) = 83.405, *p*≤0.0001] stimuli, and is reminiscent of sequential foreperiod effects [21], in which the RT on a target detection task is modulated by the time of target occurrence on the previous trial.

**Figure 4:**
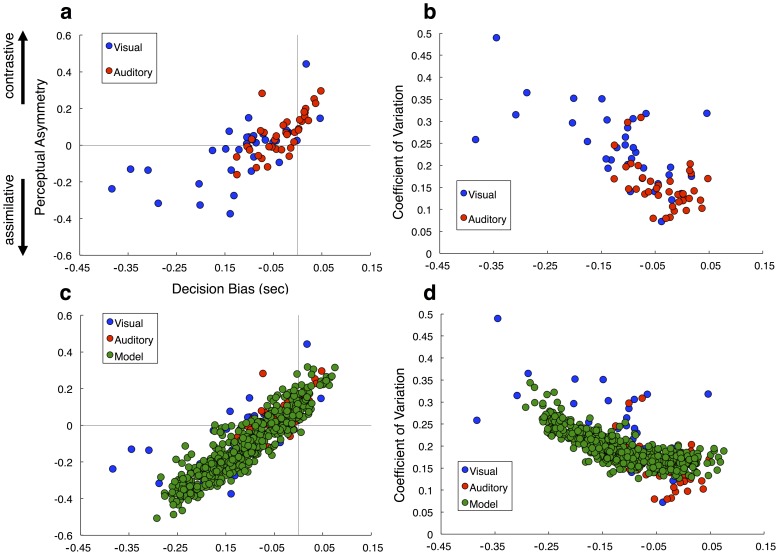
Relationship between decision bias and perceptual influence in individual participants. (**a**) Correlation between decision bias, defined as the signed difference between prior(long) and prior(short) bisection points, and perceptual asymmetry, defined as the slope of the best fitting regression line through bisection points for each prior interval condition; negative slopes indicate assimilative effects whereas positive slopes indicate contrastive effects. Smaller decision bias (closer to zero) was associated with greater contrastive perceptual effects, whereas greater decision bias (more negative) was associated with assimilative effects. Auditory and visual participants occupied separate quadrants, with greater decision bias for visual stimuli and greater perceptual contrast for auditory stimuli. (**b**) Correlation between decision bias and mean session coefficient of variation (CV); greater decision bias was associated with a larger CV. (**c, d**) Auditory and visual participant data with model performance from 500 permutations overlaid for both correlations from above. The model was able to account for a wide variety of performances that encompassed the majority of participants.

A remarkable difference between the decisional and perceptual carryover effects is that both effects shifted the position of the BP, yet in opposite predictions. However, we observed that these two effects existed to different degrees between auditory and visual stimuli; auditory stimuli engendered more perceptual carryover and less decision bias, whereas visual stimuli the opposite pattern. This difference is noteworthy, as duration was identical between the two conditions, and suggests that sensory modality has a differential impact on decision-making and adaptive mechanisms for time. In order to quantify these differences, we determined indices for both effects. Decision-bias, characterizing the effect of the prior response, was characterized as the signed difference between BPs for each prior condition [(prior *r*Long) – (prior *r*Short)]; a negative value for this index indicates that responses were assimilated by the response on the previous trial. To quantify perceptual carryover, we used the slope of the best fitting linear regression to the BP values across all seven prior interval conditions. A positive value thus represents a contrastive effect, with responses shifting away from the prior interval, whereas a negative value represents an assimilative effect. A strong correlation between the two values was exhibited [Pearson *r* = 0.711, *p*<0.0001], that also existed separately for auditory [Pearson *r* = 0.717, *p*<0.0001] and visual [Pearson *r* = 0.649, *p*<0.0001] participants alone (Figure 4a). Individual participants who showed large degrees of contrastive perceptual carryover showed lower degrees of decision bias, whereas participants with large degrees of decision bias exhibited either no perceptual carryover or an opposite, assimilative carryover. Furthermore, individual data points for auditory and visual stimuli occupied separate quadrants of the correlation, confirming our mean group findings, but also showing that the effect is continuous across individuals.

Two points are worth noting regarding the correlation between decision bias and perceptual carryover effects. First, although both indices are correlated, they are both drawn from the same dataset and both describe shifts in the bisection point; as such, when both effects travel in the same direction, they will necessarily be correlated. For example, when the previous response is “long”, it is more likely that the previous stimulus was also long in duration, and so any assimilative (or contrastive) effect measured by the decision bias and perceptual carryover indices will quantify the same shift in the BP (lower-left and upper-right quadrants of Figure 4a). However, it is noteworthy that a number of participants in our sample exhibited *both* assimilative and contrastive effects simultaneously (upper-left quadrant of Figure 4a). These participants thus exhibited both an assimilation of the current decision to the previous response, and a contrast of the perception of the current duration away from the previous duration.

In addition to the relation between perceptual and decisional carryover, we examined the influence of these indices on other main effects. The result of this analysis demonstrated that only decision bias had a strong influence on the mean session CV, manifesting as a negative correlation [Pearson *r* = −0.687, *p*<0.0001], that again existed separately for auditory [Pearson *r* = −0.469, *p*<0.005] and visual [Pearson *r* = −0.623, *p*<0.0001] participants (Figure 4b), suggesting that decision bias serves as a hidden source of variance in mean session performance.

### Computational Modeling

The behavioral findings of the present experiment exhibit a number of novel findings not previously demonstrated for duration. Moreover, these effects are unaccounted for by extant models of time perception. Current Bayesian accounts of timing judgments assumes that individuals combine noisy sensory representations with a memory prior based on the range of intervals in the stimulus set, in order to reduce uncertainty associated with timing judgments [5], [6], [22]. In our experiment, the mean of the memory prior likely serves as the criterion for categorization; however, these models assume the prior is accurately represented, and so predict no change based on recent events. Computational models investigating how the memory prior is acquired throughout a session utilize Kalman filtering, where the prior is iteratively updated after each trial [23]. In this case, an additional factor known as the Kalman gain (*g*) determines how much each new event is weighted in the next computation of the prior. If *g* declines over the session, the prior becomes stabilized and accurately represents the distribution of stimuli, but if *g* remains high, the prior is always influenced by the most recent trial [24].

**Figure 5:**
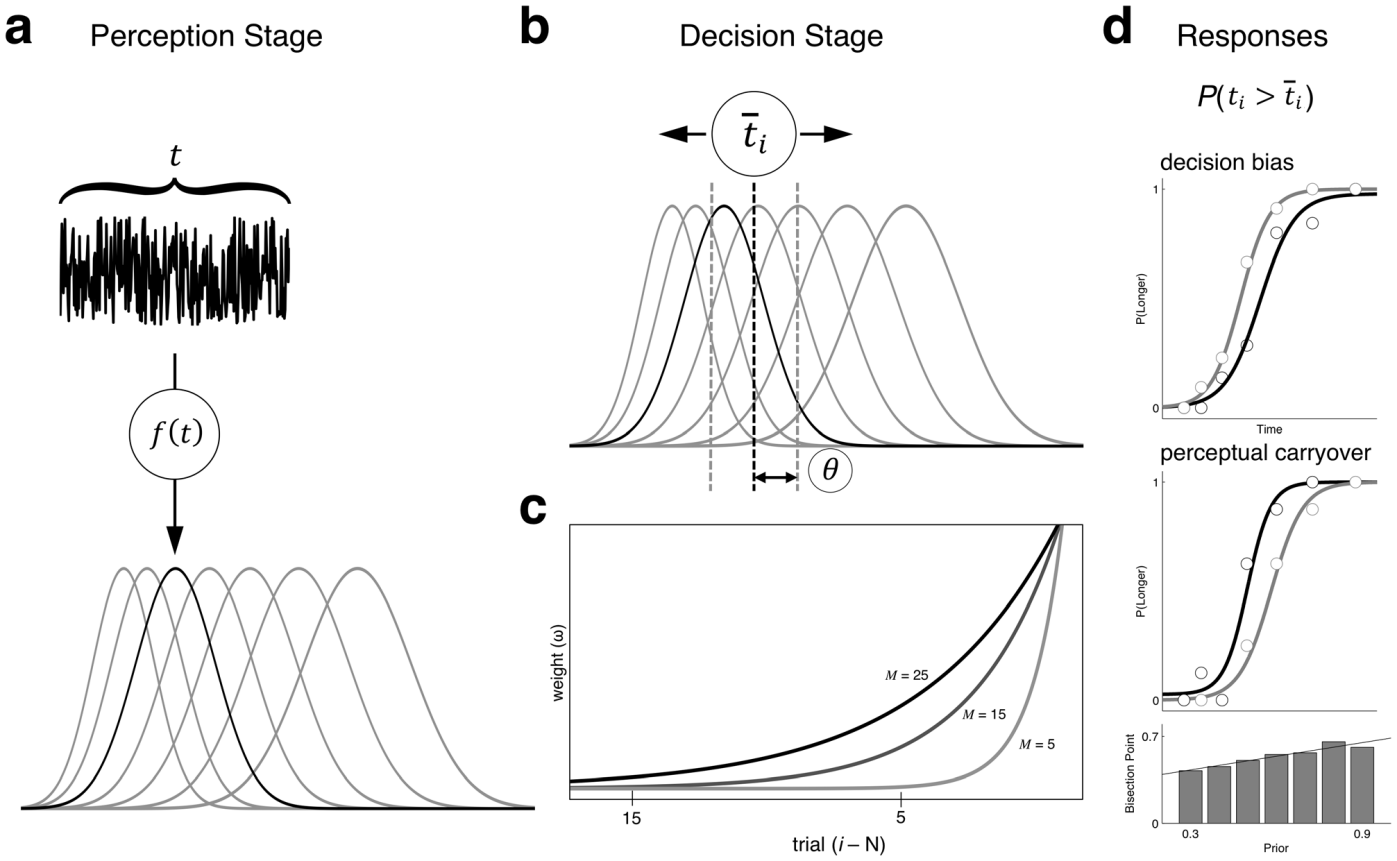
Implicit memory model schematic. (**a**) On a given trial, a temporal interval *t* (here, auditory) is perceived as a draw from a Gaussian noise distribution *f*(*t*) that varies between trials and scales with longer durations (normalized here for presentation). (**b**) Once the length of the interval is perceived, the estimate is compared to the present criterion, (black dashed line), which changes from trial to trial depending on the prior distribution of trials. An uncertainty threshold, *θ* (gray dashed lines) indicates the distance surrounding the criterion beyond which an interval can be accurately discriminated, and is also drawn from a Gaussian distribution. (**c**) The memory criterion was formed by adaptively weighting a limited number of intervals in the immediate stimulus history, limited by a given memory window size (*M*). The weighting function of preceding intervals (*ω*) exponentially decayed in time and was proportional to *M*, such that larger window sizes led to a longer decay function and a greater influence of intervals further back in the stimulus history; curves represent the decay function for different values of *M*. (**d**) The decision stage on each trial ideally categorized perceived stimuli as longer or shorter than the criterion if they exceeded uncertainty. The model reproduced patterns of decision bias (top graph: gray line  =  prior resp(shorter); black line  =  prior resp(longer)) by selecting the prior response when uncertainty could not be overcome, and exhibited perceptual carryover (middle graph: gray line  =  prior dur(900 ms); black line  =  prior dur(300 ms)) by judging each stimulus relative to the adaptive prior. Bottom graph displays model data bisection points displaying a linear (contrastive) effect of prior interval.

In order to properly account for temporal context, we developed an ideal observer model that utilizes a Bayesian heuristic approximation of behavior [18]. Specifically, we assumed that the brain continuously adapts to a fluctuating temporal context; however, we also assumed that the brain is limited in the retention of temporal information, and so implemented a “leaky” prior, in which information about past time intervals is continuously lost. As such, the memory prior only includes remembered intervals extending back for a limited number of trials (*M*). We modeled the perception of duration on each trial as a noisy sensory process, with each interval drawn from a Gaussian probability distribution with a standard deviation that grew proportionally with longer intervals, in accordance with the scalar property of time (see Methods). Next, we implemented a function of the adaptive influence of the perceived, rather than veridical, intervals within our limited memory window (figure S1a). This function, which takes the form of adaptive exponential decay, served as a weighting parameter for remembered durations, and so more recent intervals were weighted more heavily when calculating the mean of the memory prior [25] (Figure 5). In this way, our weighting function approximates a high Kalman gain. Adaptive decay functions have been demonstrated in a variety of sensory and memory phenomena [26]–[29] and so were explicitly implemented. In our model, the memory window and adaptive influence are intrinsically related; longer windows lead to a greater influence of trials further back in the stimulus history, characterized by a longer decay function.

One aspect of decision-making that most models must account for is uncertainty [30]. In our approach, we modeled uncertainty as a difference threshold limit (*θ*) that fluctuated randomly between trials with Gaussian noise, and represented the minimum temporal distance needed to accurately discriminate a temporal interval from the mean of the memory prior [31]. On a given trial *i*, the optimal observer in our model thus implements a decision rule, in which the present trial duration *t_i_*, estimated with sensory noise, is compared to the current value of the adaptive prior outlined above; if the difference between these two values exceeds *θ*, then the observer responds ideally with the correct decision. If the uncertainty threshold is not reached, then the observer selects the same response as on the previous trial (*i*–1). Previous work has demonstrated that, when provided feedback on each trial, subjects will respond similarly to the reinforced choice on the previous trial when the perception of the current trial is ambiguous; without feedback, subjects will respond similarly to the previous response [20]. An implication of this finding is that the previous response represents the “best guess” when the current stimulus identity is uncertain, which may arise from the finding that human subjects are naturally biased to perceive positive correlations between randomly associated values [26]. In this way, uncertain estimates are chosen in a manner that is non-random, and depends on the prior stimulus decision history. Furthermore, the adaptive nature of the prior allows for shifts in uncertainty for the same temporal interval that is directly proportional to shifts in the prior. For example, a 433 ms interval is more likely to be estimated with uncertainty when the mean of the prior is near 433 ms, but will become less uncertain as the prior shifts away.

In order to compare the performance of our observer model to behavioral data, we ran 500 simulations with the window size of the memory prior (*M*) and the uncertainty threshold (*θ*) as the only free parameters, being randomly assigned for each permutation (see Materials and Methods). Variability of the sensory estimates was constant, at a value that matched the mean variability of auditory participant estimates (CV = 0.16). We chose a single value for the CV for several reasons. First, we wanted to reduce the number of free parameters in our model; second, our value of CV matches previous estimates of the CV for temporal bisection performance across a wide variety of studies [16]; third, we wanted to see if carryover effects alone could account for timing variability, without adding noise to the estimation process. That is, although participants exhibited CVs higher than 0.16, we wanted to see if, by modifying carryover effects, we could account for greater session variability. We found that both decision bias and perceptual carryover effects were recapitulated in our modeled dataset, with a range of values that were strikingly similar to the values of participants (Figure 4c). The similarity between the simulated and observed data suggests that human participants adopt a strategy that is in line with the Bayesian heuristic implemented in our model. Also, it is noteworthy that our model was able to reproduce the pattern of simultaneous assimilation for decision bias and contrast for perceptual carryover observed in human participants (upper left quadrant, Figure 4c). Additionally, we found that our model exhibited the same correlation between decision bias and variability as in human participants (Figure 4d). This is noteworthy as sensory variability was set in our model at a constant value that did not vary between simulations. As such, variability in decision bias alone could account for an increase in CV over twice the size of modeled sensory variance, suggesting that the CV is highly susceptible to decision bias.

**Figure 6:**
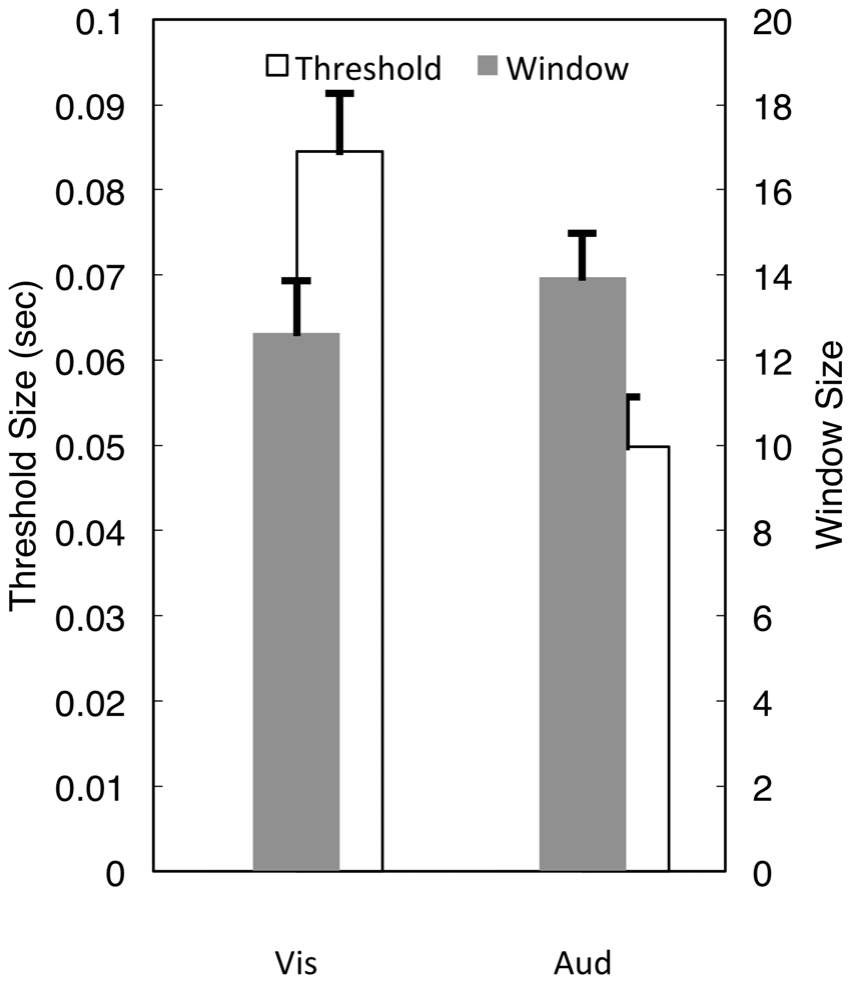
Model fits of experimental data. Individual fits indicated that the window size of the memory prior did not differ between participants performing on the auditory or visual versions of the task. However, uncertainty threshold values did significantly differ, with higher thresholds for visual participants (*p*<0.05). Error bars represent s.e. of the mean.

In order to address the contribution of *M* and *θ* parameters to participant performance, we fit our observer model to individual participants (Materials and Methods, Figure 6). Our model fits indicated that both visual and auditory participants were able to hold a similar number of intervals in the memory prior, (∼13 intervals), with no significant difference between the two groups [*t*(71) = 0.815, *p* = 0.418]. For the uncertainty threshold *θ*, we found that visual participants exhibited a significantly higher mean threshold (84 ms) than auditory participants (49 ms) [*t*(71) = −3.842, *p*<0.0005], suggesting that a crucial difference between auditory and visual timing is the uncertainty associated with judgment comparisons, rather than the fidelity of the sensory system for independently estimating a given duration.

We further note that our model exhibited a number of patterns not explicitly modeled. Foremost, we found that duration estimates exhibited the classic pattern of central tendency, with a regression to the mean (figure S2), suggesting that short-term dependencies can give rise to the pattern of central tendency [31], [32], and that central tendency can arise from a simple adaptive process, rather than an optimal reduction of noise that is seen as separate from carryover effects [26]. Additionally, we found that the fluctuating memory prior exhibited 1/f noise characteristics (figure S1b), a commonly observed feature in models of memory, which further suggests that long-term patterns can arise from a limited number of trials [32].

### Model Variations

Although our model was able to recapitulate the carryover effects observed in human participants, it is possible that these effects did not rely on the entire model as designed. That is, it is unclear how much each assumption in our model contributes to the observed data. In order to measure the influence of particular factors, we constructed four alternative versions of our model (see Materials and Methods). In the first alternative, we tested the influence of the limited memory prior by generating an ideal observer with an unlimited memory (unlimited-prior model). In this version, recent trials are still weighted more heavily, but every trial is retained in the memory prior. As the number of trials retained in memory shapes the adaptive decay function in our model, the influence of prior trials decays much more gradually. In the second alternative, we tested the influence of the uncertainty threshold *θ* by generating an observer with zero uncertainty (zero-uncertainty model). In this version, the observer can always identify if the current interval is longer or shorter than the mean of the memory prior, and so never intentionally responds similarly to the previous trial. In the third alternative, we tested the influence of the adaptive decay function by removing its influence entirely (zero-weighting model). In this version, the observer still retains only a limited number of trials in memory, but there is no weighting of more recent trials. In the fourth alternative, we asked if the uncertainty threshold and the implicit memory prior are computed independently, or rather mutually depend on the statistics of the environment. Recent studies have shown that both animals [33] and humans [25] can adapt to the variability of temporal information when the intervals tested are unknown, but stable. Whereas the prior is derived from the mean of the durations stored in memory, it is possible that the threshold is derived from the variance of remembered durations. In the fourth version, the width of the Gaussian distribution used for drawing *θ* for each trial was determined by the variance of intervals stored in memory (memory-based uncertainty). In this way, the uncertainty in any judgment between a current interval and the mean of the prior depends on both the variance of the prior and the number of the trials retained. Furthermore, memory distributions retaining a larger number of repeats will also be characterized by less variance [34].

**Figure 7:**
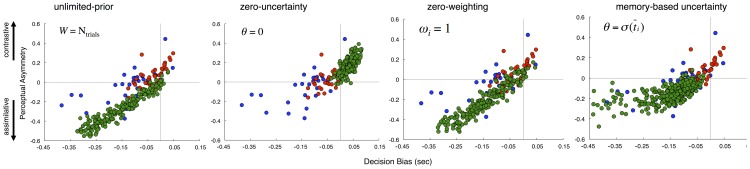
Alternative model comparisons. Each graph displays the results of 200 permutations of an alternative model (green points) compared with the original model (faded points). Four models were tested: an unlimited-prior model, which assumed a perfect memory prior that continuously integrated all perceived intervals across the entire session; a zero-uncertainty model, where uncertainty in the comparison of the present duration with the mean of the prior did not impact decisions and *θ* was set to zero; a zero-weighting model, in which the prior was still limited and varied between permutations, but no adaptation weighting was applied (all remembered intervals contributed equally to the prior); a memory-based uncertainty model, where *θ* was set to match the variability of the intervals stored in the limited memory prior. None of the alternative models reproduced the full pattern of data observed in participants or in the full model.

Comparison of the alternative models with the original data is displayed in Figure 7 and figure S3. Notably, none of the four models covers the same range of data as available in the full implementation. Comparison of the fits to subject data between the original model and alternative models demonstrated that the original model fit the data significantly better than any of the alternatives (all *p*<0.05; table S1). However, we do note that, when segregating the data by modality, the memory-based uncertainty model and the original model fit the data from visual-modality subjects equally well [*t*(35) = 1.182, *p* = 0.245]; we additionally note that the zero-weighting model fit the data for both auditory and visual participants quite well, with the original model only being better at the trend level (*p* = 0.058; table S1). However, the alternative models allow for a dissection of the relative contribution of each parameter to both carryover effects. In the unlimited-prior model, little to no contrastive perceptual carryover is observed, with only decision bias and assimilative carryover existing. In the zero-uncertainty model, the perceptual carryover effect dominates observer responses, with most observers showing little to no decision bias, and contrastive perceptual effects dominating. Comparing values of *M* and *θ* between both models revealed that veridical performance, and very little carryover, is associated with high values of *M* and low values of *θ* (figure S4). The zero-weighting model shows only a slight increase in contrastive carryover, indicating that this effect likely relies on an adaptive decay that is limited in time; however, this model was still very successful at fitting the data, suggesting that a limited window of trials is also important for producing carryover. Lastly, and perhaps most importantly, the memory-based uncertainty model exhibited only assimilative biases, suggesting that, for both assimilative and contrastive effects to occur in decision bias and perceptual carryover, the width of the uncertainty distribution must be computed independently of the memory distribution. We also note that none of the alternative models were able to fully reproduce the simultaneous pattern of assimilative and contrastive effects observed in the full model (upper left quadrants of Figure 7).

As a final test, we also examined a variation of our model in which the scaling factor for variability of the perceptual measurement of each duration (*t_σ_* in equation 1) was also set as a free parameter. We found that this additional model also exhibited the same carryover effects as when variability was held constant, in our original model, and fits to individual participants produced equivalent of *M* and *θ* as the original model (figure S7). However, we also found that the fits to auditory and visual participants produced variability values that were not significantly different from one another [*t*(69) = -0.592, *p* = 0.556], further suggesting that a crucial difference between the auditory and visual modalities for time is the threshold for measuring differences, rather than the noisiness of measuring intervals.

## Discussion

The main finding of our study is that human participants exhibit serial dependencies in temporal bisection. These dependencies take the form of decision bias, in which the current response is assimilated by the response on the previous trial, and perceptual carryover, in which the perceived duration of the current stimulus is contrasted away from the previous duration. Notably, each type of carryover effect pushes responses in opposite directions, and can even occur simultaneously in some individuals. These findings are rendered invisible when looking at the mean level of performance across a given session. However, mean variability (CV) is strongly influenced by the degree of decision bias in our sample, suggesting that decision-level variance and perceptual variance can be dissociated into separable components.

In addition to the present findings, we note that our model can accommodate a diverse set of findings in temporal perception. Foremost is the observation that our model replicates measures of central tendency, in which estimates of duration are systematically biased toward the mean of the stimulus distribution. Previous models have suggested that central tendency arises through an optimal Bayesian integration process that improves precision [5]. Crucial to these models is the assumption that the observer has access to an accurate representation of the prior. Although the shape of this distribution may change, depending on stimulus features and the shape of the underlying distribution [6], [35], it is generally thought that participants accurately learn the range of the prior relatively early in the session [24]. However, this formulation precludes the occurrence of carryover effects, except for early in the session when the prior is learned [23]. In our model, participants have access to a representation of the prior that is both limited in time and adapted by recent trials. Notably, there is no assumption in our model that the prior reduces variability; rather, the uncertainty threshold that surrounds the prior is where changes in variability arise.

A second type of finding in temporal perception that can be explained by our model is anchor effects. A number of studies have now demonstrated that the response to a stimulus can depend on the presentation of previous intervals that are irrelevant to the required judgment [9], [36]–[38]. Our model can predict such effects by assuming that previous intervals are integrated into the adaptive prior, such that their influence still lingers in the judgment of a subsequent duration. That is, even if the task requires a participant to discriminate between two temporal intervals, previous intervals will interfere with that judgment by influencing the memory for duration.

### Context dependent and independent timing

Recently, there has been debate about the role of context dependent versus independent representations of time in the human brain [1], [2]. Context dependencies extend beyond the distribution of intervals, and include the sensory modality and motor requirements of the task. Recent research has shown that different neural circuits are invoked, depending on the temporal context [39], suggesting that different circuits are optimal for different contexts. Our findings suggest that different modalities are susceptible to different kinds of contextual carryover effects; auditory stimuli exhibit more perceptual carryover, whereas visual stimuli are more susceptible to decision bias. These findings are in accordance with recent evidence suggesting that auditory stimuli engender the rapid formation of representations [40] and that A1 neurons are susceptible to prior stimulus history [28] while attention to visual stimuli impairs performance with nearby distractors [41]. Furthermore, these findings support theories suggesting that stimulus encoding relies on a relative, rather than absolute, coding scale, obviating the need for long-term representations [42].

Regarding the neural basis of these effects, a number of candidate regions that have been implicated in temporal processing may be related. Electrophysiological recordings have implicated inferior parietal neurons in the encoding of temporal information leading to a decision in a task similar to that used here [43]. Additionally, transcranial magnetic stimulation of inferior parietal cortex in humans has been found to shift perceived duration, independent of response bias, and increase activity at frontocentral sites [44]. Other recent research has demonstrated that supplementary motor area (SMA) and basal ganglia neurons exhibit tuning preferences for different durations [2], suggesting that the SMA and basal ganglia may serve as an accumulator-readout for time. However, it may be that perceptual carryover and decision bias are based within separate neuronal populations in distinct regions.

### Adaptive Timing

Recent research has shown that adaptation-level effects for a variety of stimulus attributes can alter perceived duration [36], [45], [46]. Here we extend these findings by showing that time itself can adaptively change perceived duration. This finding is consistent with a recent channel-based model of time that showed adaptation effects for long exposures of repeated temporal intervals [3]. Regarding the neural basis for such effects, one possibility is that incoming sensory signals encode temporal duration as a basic stimulus feature. Recent research has shown that V1 neurons can learn to encode temporal expectancies [47], and that A1 neurons can encode temporal rate information [48]. Temporal information may thus be encoded by the detection of offset responses that are temporally tuned for a range of intervals [2].

Adaptive timing may be advantageous for organisms by allowing for the formation of temporal expectancies. Indeed, a major challenge for the nervous system is making predictable inferences in the face of dynamic, noisy stimulus environments; adaptation to time may thus allow the nervous system to predict when particular events are going to occur and thus maximize information transmission [49]. Recent research has shown that forming accurate temporal expectations improves behavioral performance and the signal-to-noise ratio of sensory information [50], which may relate to dynamic changes in sensory cortical neurons [47], [51]. Our findings suggest a model of adaptation that responds to a perceptually stochastic stream of intervals by attempting to home in on a mean criterion.

An additional mechanism that may be invoked in adaptation is repetition suppression [52]. Indeed, recent suggestions have been made that time perception may be mediated by the strength of neural firing for stimulus features. In these energy-readout models, variations in perceived duration arise from the repetition of stimulus features [36], such that the repeated pairing of two stimuli of equal durations leads to a relative contraction of the duration of the second stimulus relative to the first. Although not specifically hypothesized within our model, we explored the possibility that repetition of stimulus duration leads to a change in perception, and so may have contributed to our findings. However, we found no change in the perceived length of duration when the same interval was repeated, measured relative to the null condition, where the preceding trial was a blank screen, and so no adaptation should have occurred (Figure S5). This finding suggests that the changes we observed were not due to differences arising from a repetition-suppression type mechanism for duration.

In the present study, we utilized a path-guided de Bruijn sequence to test for the effects of carryover. The use of the de Bruijn sequence in cognitive neuroscience was originally intended for fMRI studies as a test of continuous carryover of stimulus attributes. Accordingly, measures of carryover in fMRI BOLD signal are related by the difference between successive pairings of stimuli; a difference in signal intensity changes that matches the presumed distance between pairings can be used to isolate neuronal populations that are tuned for the manipulated stimulus attribute. The primary advantage of this technique is that the results of the present experiment may be easily adapted for neuroimaging. Furthermore, computational modeling of prior duration, uncertainty and adaptive weighting may all be applied to model-based fMRI designs [53]. Lastly, we note that our paradigm may be useful for developmental, psychiatric and neurological studies of patient populations. Numerous timing deficits in psychiatric and neurological pathologies have been previously identified [54], and developmental studies have tracked how timing abilities normally or abnormally develop [55]; notably, many of these studies utilize the temporal bisection task for identifying timing disturbances. Additionally, a number of these studies find disturbances or changes in variability, characterized by larger CVs. Changes in the CV have previously been interpreted to reflect alterations in the underlying representation of temporal intervals. In our study, we demonstrate that CV is affected by decision bias, rather than the underlying representation. By utilizing our design for temporal bisection, it is possible to measure perceptual carryover and decision bias effects, and so provide a more nuanced profile of timing deficits and differences in different populations.

Lastly, in our model, we formulated the representation of time on a given trial as a noisy process that is normally distributed and scales with duration length, approximating a likelihood or basis function [5]. However, we leave it open how this representation is generated. Accordingly, our model could be integrated into any number of available models for timing that incorporate scalar variability. We suggest that a likely candidate for the creation of sensory representations could be found in a recent drift-diffusion firing rate model of time perception [10]. Our reasoning is that drift-diffusion models are well suited for predicting behavior on 2AFC paradigms, including the shape of the chronometric function observed in the present study [19]. We additionally note that our model as it applies to the current dataset leaves open other refinements. Particularly, we modeled uncertainty and prior size parameters (*θ* and *M*) as stationary across a given session. In practice, this is unlikely, as non-stationarity is a common occurrence in psychophysics [12]. One possibility may be that *θ* and *M* adaptively change throughout a session to periods of perceptual stability; such an implementation would be similar to the Bayesian heuristic solution for “change-point” processing, wherein the set of presented stimuli randomly shifts throughout a session [56]. Additionally, we note that the memory prior in our model does not include the ITI between trials as a factor. Longer delays between trials may be associated with greater decay in memory, and changing ITI length has previously been shown to influence carryover effects for other stimulus dimensions [57]. Indeed, in our model, we modeled decay across trials, rather than time, and further assumed that the memory window is limited to a particular number of trials. Yet, there is no reason why our model could not be implemented with a memory process that decays over time, or one that does not require a memory window but instead has the weight of distant intervals decay practically to zero. In either case, we expect the results of such models to be similar to the one presented here. However, future implementations of our model may require adjusting these and other variables throughout a session.

We further note that our model of human performance shares a number of commonalities with other models of timing, such as memory mixing [58], [59], uncertainty thresholds [31], and implicit standards [25]. However, we note that none of these other models in their current forms can accommodate the set of findings in our data; that is, in order for a model to apply to trial-by-trial carryover effects, it must produce *both* decision bias and perceptual carryover *simultaneously*. Our choice to compare our model to alternative variations represents an attempt to explore the contributions of each of our assumptions. As shown, none of our model alternatives were able to fully account for the data, suggesting that carryover effects require both a computation of uncertainty and an implicit memory prior that is adapted in time. Of most interest in this regard are the results of our fourth model alternative, where the degree of uncertainty is determined by the variability of the environment. Although it has been demonstrated in other timing domains that human and animal participants account for the variability of time intervals across a given session [33], [34], our results demonstrate that this information is not used for measuring uncertainty in the estimates. This finding suggests that uncertainty is calculated in the brain separately from memory variance. It is also interesting then to note that when fitting the model to individual participants, we found larger values of *θ* for visual than auditory participants, whereas the size of the memory prior was equal between modalities. The implication of this finding is that uncertainty is calculated in modal-specific regions, whereas memory for duration is stored amodally.

Finally, we note that our investigation and modeling of carryover effects has implications beyond time perception. Indeed, carryover effects are a long-standing psychological phenomenon that have been demonstrated for a variety of stimulus properties, such as pitch [57], size [14], density [60] and brightness [61], as well as more complex stimuli such as faces [62] and monetary value [63]. We suggest that our findings and model may thus generalize to the larger corpus of carryover effects and can provide some insight.

## Conclusions

Studies of duration processing entail an understanding of the temporal context in which intervals are presented. The results of the present study demonstrate that contextual effects extend to single-trial data, where the duration and response choice of a given trial exerts a diminishing influence on future trials. Our findings suggest that time is an adaptable attribute of stimulus encoding that adjusts to dynamic changes in unpredictable stimulus contexts.

## Materials and Methods

### Ethics Statement

Written informed consent was obtained from all subjects, and the Institutional Review Boards of both the University of Pennsylvania and George Mason University approved the study protocol.

### Participants

A total of 80 right-handed participants, age 18–30 years, were included. All participants were naïve to the task design and no participant performed in any more than one version of the task. Participants were drawn from students around the University of Pennsylvania and George Mason University campuses, and were assigned course credit for their participation.

### Stimuli and design

Participants performed a modified version the temporal bisection task (partition variant; [15]). All participants sat in front of a gamma-corrected, cathode-ray tube (CRT) monitor, with a refresh rate of 100 Hz. Participants viewed a series of stimuli, one-at-a-time, that persisted for one of seven logarithmically spaced intervals of time, between 300 and 900 ms. On each trial, participants were required to judge whether the stimulus presented was “long” or “short”, based on their own subjective feeling, and press one of two response keys for each choice. Participants were instructed to make each response as quickly, yet as accurately as possible, and not to over-think their responses. At the beginning of the experiment, participants were presented with three stimuli at the geometric mean of the stimulus set as an example of the average stimulus duration and for comparison purposes for the first few trials. All visual stimuli were generated in the Python programming environment using extensions provided by Psychopy, version 1.75 [64], and consisted of a centrally-presented Gaussian luminance blur, presented at 100% contrast against a grey background (mean luminance: 117 cd/m^2^) with a FWHM of 2 cm. All auditory stimuli were generated using Audacity, version 2.0 (http://audacity.sourceforge.net/), and consisted of a white noise burst (0.5 amplitude, 44100 Hz digitization), presented via headphones at a comfortable volume, individually adjusted for each participant (loudness range: [69–73 dB). Each trial consisted of the presentation of a centrally presented fixation point for 500 ms, followed by the presentation of the stimulus of variable duration, followed by a blank screen that was terminated by a choice response. The order of stimulus presentation was determined by a path-guided de Bruijn sequence (https://cfn.upenn.edu/aguirre/wiki/public:de_bruijn). de Bruijn sequences are modified Hamiltonian cycles through a stimulus set, such that every possible order combination of stimuli is presented [65]. The path-guided process of the de Bruijn sequence allows the Hamiltonian cycle to be modified by a guide function, which can provide an underlying structure to the perceptually stochastic sequence in which stimuli are presented. The guide-function was modulated by a sum of sinusoids with random periods between 20 and 40 elements (unit labels). An additional label for null (empty) trials was added to the matrix, so as to include trials where no stimulus was presented; on null trials, subjects viewed a blank screen for 550 ms, followed by the appearance of the fixation point for the next trial. The resulting trial matrix consisted of 64 possible trial types and a sequence of 512 trials. Each duration in the total sequence was presented 64 times. For each of the prior conditions, each duration was presented eight times.

### Behavioral Analysis

The first trial was removed for each participant from the analysis. All trials were additionally filtered by a RT cutoff of 1000 ms, such that trials for which the RT exceeded 1000 ms were discarded (figure S6); we chose this threshold to limit ISI length and on the basis of work suggesting that serial dependencies are strongest for ISIs within this range [66]. Psychometric and Chronometric curves were first generated for each participant based on the full dataset. Psychometric curves were generated by plotting the proportion of long response choices for each of the seven tested durations; these points were then fitted by a sigmoidal, logistic curve using the *psignifit* version 2.5.6 software package (see http://bootstrap-software.org/psignifit/) for Matlab, which implements the maximum-likelihood method described by Wichmann & Hill [67]. Upper and lower thresholds, the approximate points at which the subject is 25% or 75% likely to judge the stimulus as long, were calculated using the bias-corrected bootstrap method implemented by psignifit, based on 1999 simulations [68]. The results of this analysis yielded the bisection point (BP; the time value at which subjects were equally likely to judge the stimulus as long or short), the difference limen (DL; the difference between the upper [75%] and lower [25%] threshold values divided in half), and the coefficient of variation (CV; DL/BP). The BP thus reflects the subjective midpoint of the range of tested durations, while the CV reflects the normalized variability of measurements. We note that our choice of nomenclature here reflects standard use among studies of time perception; BP is used here instead of the more common point of subjective equality (PSE), as the latter term implies an explicit comparison between two stimuli. Chronometric curves were constructed by plotting the RT for each of the seven possible durations. Participants with poor Psychometric fits were removed from the analysis; all subjects with a CV value of 1 or greater were removed, resulting in four subjects removed from the visual version of the present experiment and one subject removed from the auditory version.

For the exploration of carryover effects, trial types were divided up for each participant. To explore decision bias effects, participant responses were segregated into trials preceded by a long or short response choice, resulting in two separate conditions for each duration trial type. Psychometric and Chronometric curves were again generated using the procedure outlined above. For perceptual carryover effects, participant responses were segregated into trials preceded by each of the eight possible prior trial types. A total of eight Psychometric and Chronometric curves were generated for each of the carryover conditions.

### Computational Modeling

In order to quantify the influence of prior decision and perceptual carryover effects observed in behavioral data, we constructed an optimal observer model that implemented a heuristic Bayesian approximation of behavior. The model begins by assuming that the length of time of any given interval *t* on trial *i*, is drawn from a noisy sensory representation of the form
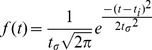
(1)which implements a Gaussian probability distribution for elapsing intervals of time *t* with mean *t_i_* and standard deviation *t_σ_*, such that *t_σ_* scales linearly with a constant coefficient of variation, in accordance with the scalar property of time and Weber's law. For modeling purposes, the CV was set at the mean for participant performance on the auditory version of our task (0.16). The model further assumes that on any given trial *i*, the observer computes a running weighted average of the bisection point, representing the midpoint of the range of previously experienced stimuli. We note that the estimate of the bisection point on each trial here represents the average of perceived, not actual, durations experienced; as such, the bisection point is based on the noisy sensory estimates of duration from previous trials. The running average for trial *i* is referred to as and takes the form
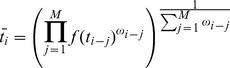
(2)where *M* is the size of the running average window and *ω* is an adaptive weighting function. The form of *ω* was modeled as an exponential decay function 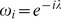
(3)where 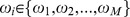 and 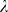 represents the decay constant with 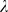 = 1/*M,* in order to more closely represent longer decay curves observed in previous studies [18], [25]–[28]. In this way, *ω* is directly scaled by *M*, so that larger windows sizes lead to longer decays. Equation 2 thus computes the weighted geometric mean of preceding temporal intervals extending back for a finite window in time, and approximates the assumed prior distribution of intervals on a given trial *i*. On a given trial, the model thus implements the following decision rule
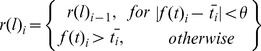
(4)where *r(l)_i_* is the response choice for “long” on trial *i* and *θ* represents an uncertainty threshold. We note that equation 4 can be altered for “short” response choices *r(s)_i_* by changing the second line to *f(t)_i_*<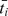. The value of *θ* represents the least discriminable interval of time, such that intervals that have a difference lower than this value cannot be accurately differentiated. We modeled this difference by drawing from a Gaussian distribution on each trial, with a mean of 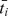 and a SD of *θ*, and then calculating the absolute difference between that value and 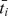. The model thus computes the difference between the presently perceived interval of time, drawn from a noisy distribution (equation 1), and the subjective midpoint of previously experienced intervals, drawn from the prior distribution that is limited in time and weighted by recent adaptation (equation 2). If the difference between these two values exceeds the uncertainty threshold (*θ*), then the subject responds ideally by indicating whether *t_i_* is longer or shorter than 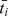, otherwise, if the difference does not exceed uncertainty, the observer simply responds with the same response from the previous trial *i*–1.

Monte Carlo simulations of the observer model were run through the de Bruijn sequence of presented stimuli; with *M* and *θ* as the only two free parameters. Values of *M* ranged between 1 and 30 (step size = 1), whereas *θ* ranged between 1 and 150 ms (step size = 1 ms). For a given run of the model, individual responses were concatenated and analyzed with the exact same method as the behavioral data outlined above. Model permutations were run 500 times, with eleven psychometric curves generated for each run, giving a total of 5500 psychometric curves. Decision bias and perceptual carryover effects were evaluated in the same manner as behavioral data (see Results) for each run.

In addition to the original model, four model variants were also run to test for the contribution of any given parameter to the model: a zero-uncertainty model, in which the value of *θ* was set to 0, and so no prior decisions were ever selected; an unlimited-prior model, in which the value of *M* grew proportional to the number of trials tested, ensuring that no intervals were ever lost from the prior; a zero-adaptation model, in which the value of *ω* was constant, so that no preferential weighting of prior intervals occurred. Lastly, we contructed an alternative model where the value of *θ* was determined by the SD of intervals in memory; in this model, *θ* was calculated on each trial as the standard error of 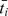. Each of these models were permuted 200 times for comparison with the results of the main model.

We fit the original model to individual participant performance by using the values of *M* and *θ* from our original Monte Carlo simulation. This was accomplished by calculating the root mean-squared error (RMSE) between predictions of the model and human performance [33]. As participants each exhibited decision-bias and perceptual carryover, we calculated the RMSE between model outputs and these values for each subject [16]. The model output that best matched human performance was found by finding which model parameters out of the permuted set had the minimal RMSE for each participant. Alternative models were fit in the same manner to participant data, and RMSE values were compared between models. However, we acknowledge that this comparison is limited, as not all of the alternative models had the same number of free parameters.

### Data Availability

The behavioral data and computational model from this study are both freely available online at http://archlab.gmu.edu/pang/resources.html.

## Supporting Information

Figure S1 **(a) Example run of the observer model.** Black circles represent the perceived (not actual) duration on each trial; green shadings represent the noisy distributions from which the perceived duration was drawn. The red line represents the weighted geometric mean of perceived durations in the implicit memory prior. (**b**) 1/f scaling in model performance. Left columns display the evolving value of the criterion for three modeled observers with decreasing window sizes. Right columns display the corresponding power spectra in log space. As the window size for the memory prior increases, the power spectra exhibit a greater linear decline in power, consistent with 1/f pink noise spectra.
(TIFF)

Figure S2 **Central tendency effects of modeled data.** For each modeled participant, the mean estimate of each current duration category was calculated. The horizontal dashed line indicates the mean stimulus duration; the diagonal dashed identity line represents veridical performance. Large plotted points represent the mean of modeled observers (*N* = 500), with faded points representing individual observers.
(TIFF)

Figure S3 **Evaluation of average model fits for the original and alternative models.** Left panel displays model performance for the aggregate total; right panel displays auditory and visual data separately. Error bars represent standard error. The values plotted are [1 – RMSE], such that higher values represent better fits.
(TIFF)

Figure S4 **Comparison of model parameters between two alternative models.** In the unlimited-prior and zero-uncertainty models, only one parameter, *θ* or *M*, was varied. Colored points display the value of the varied parameter. In the unlimited-prior model, lower values of *θ* (in seconds) are associated with less carryover, whereas in the zero-uncertainty model, higher values if *M* (in trials) lead to less carryover.
(TIFF)

Figure S5 **Repetition effects.** For visual and auditory participants, grand-averaged psychometric curves are displayed representing the conditions where the same interval was presented twice (Prior Repeat), or the prior interval was a Null event, where participants viewed a blank screen. No differences in the bisection point were observed for either condition in either modality.
(TIFF)

Figure S6 **Number of trials removed after RT cutoff of 1000 ms for each duration (max = 64).** Significantly more trials were removed for visual than auditory stimuli [*F*(6,384) = 2.930, *p* = 0.008]. Both modalities demonstrated an effect of duration [*F*(6,384) = 15.688, p<0.001], with more trials being removed around the middle range of durations.
(TIFF)

Figure S7 **Fits to individual participant data of the original model with perceptual variability as a free parameter.** Model fits are shown next to the corresponding fit values from the original model from Figure 6. The model with variability as a free parameter (“Changing CV”) produced values of window size (**a**) and threshold size (**b**) that were not significantly different from their counterparts in the original model (all *p*>0.05), and also produced threshold values that were significantly higher for visual than auditory participants (*p*<0.05). (**c**) Additionally, no difference between the variability of perceptual measurements was found for fits to auditory and visual participants (*p*>0.05).
(TIFF)

Table S1 **Paired t-test values, displayed as** ***t*** **-statistics, comparing the original model fits against each of the alternative model fits.** Separate comparisons are displayed for the entire set of subjects, and separated between auditory and visual subjects.
(TIFF)
