# Supplementary figures and images for: Continuous Carryover of Temporal Context Dissociates Response Bias from Perceptual Influence for Duration
Source: PLoS One. 2014 Jun 25;9(6):e100803. doi: 10.1371/journal.pone.0100803 (PMC4071004; doi:10.1371/journal.pone.0100803)

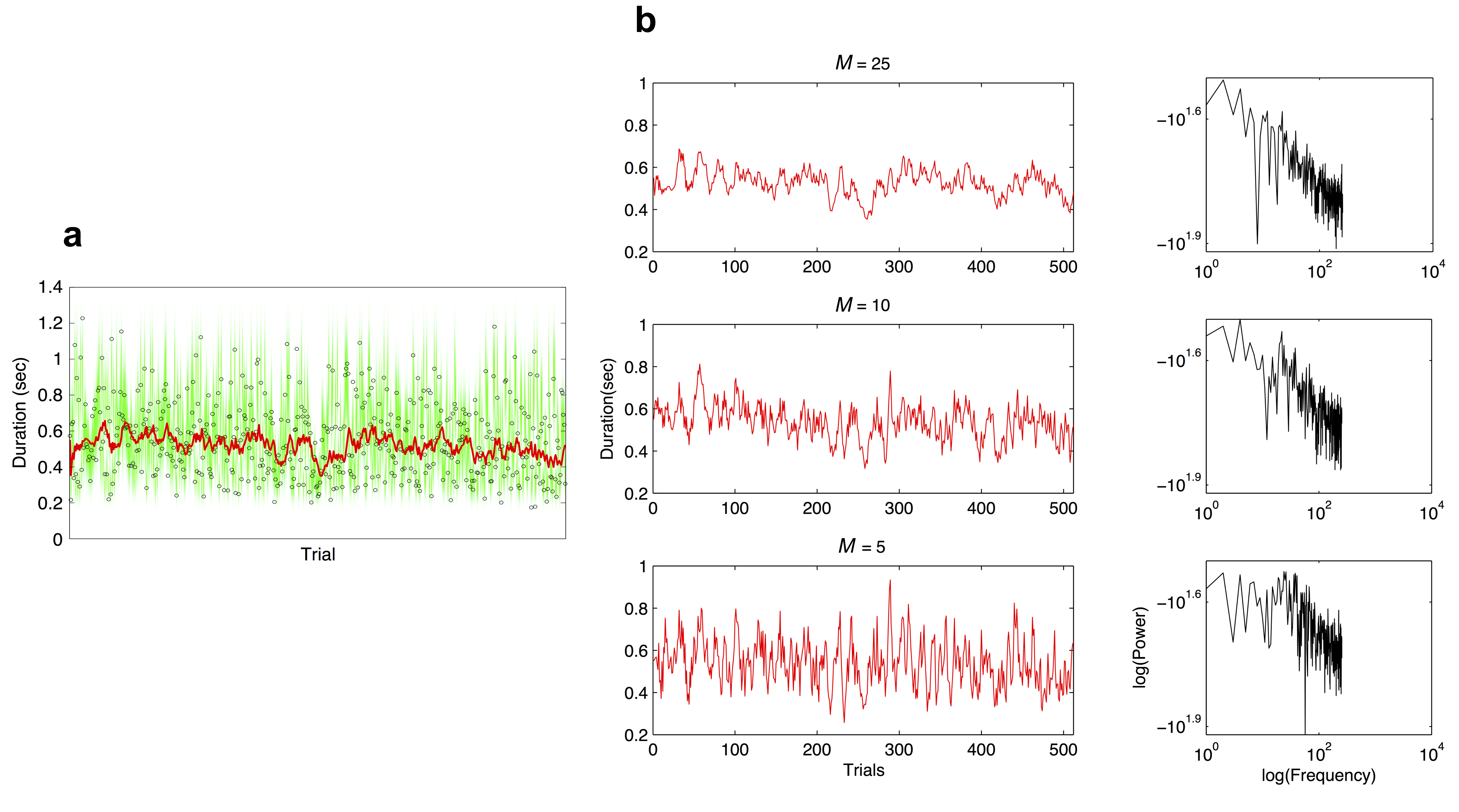

Supplement: Figure S1 — (a) Example run of the observer model. Black circles represent the perceived (not actual) duration on each trial; green shadings represent the noisy distributions from which the perceived duration was drawn. The red line represents the weighted geometric mean of perceived durations in the implicit memory prior. (b) 1/f scaling in model performance. Left columns display the evolving value of the criterion for three modeled observers with decreasing window sizes. Right columns display the corresponding power spectra in log space. As the window size for the memory prior increases, the power spectra exhibit a greater linear decline in power, consistent with 1/f pink noise spectra. (TIFF) [file pone.0100803.s001.tiff]

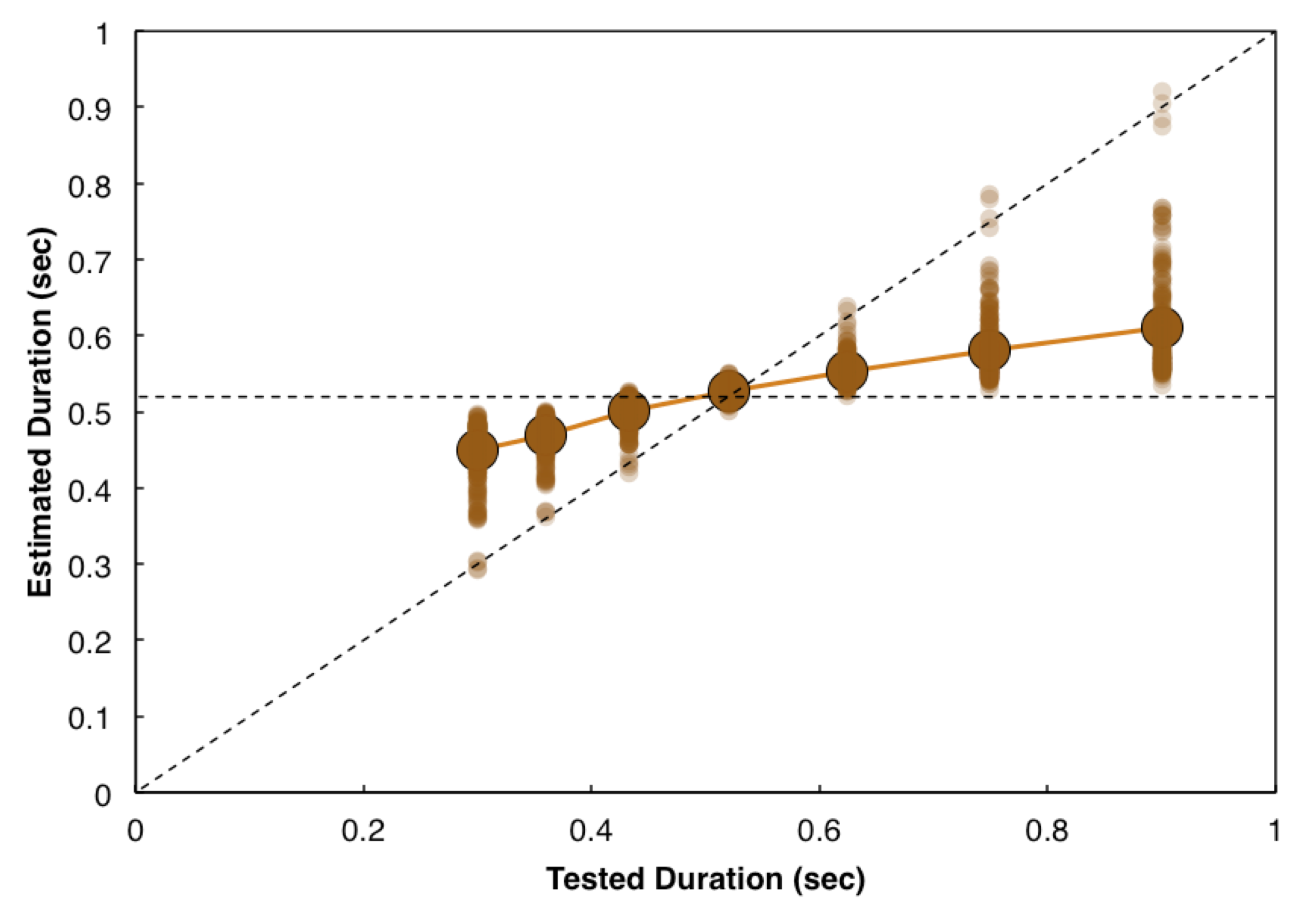

Supplement: Figure S2 — Central tendency effects of modeled data. For each modeled participant, the mean estimate of each current duration category was calculated. The horizontal dashed line indicates the mean stimulus duration; the diagonal dashed identity line represents veridical performance. Large plotted points represent the mean of modeled observers (N = 500), with faded points representing individual observers. (TIFF) [file pone.0100803.s002.tiff]

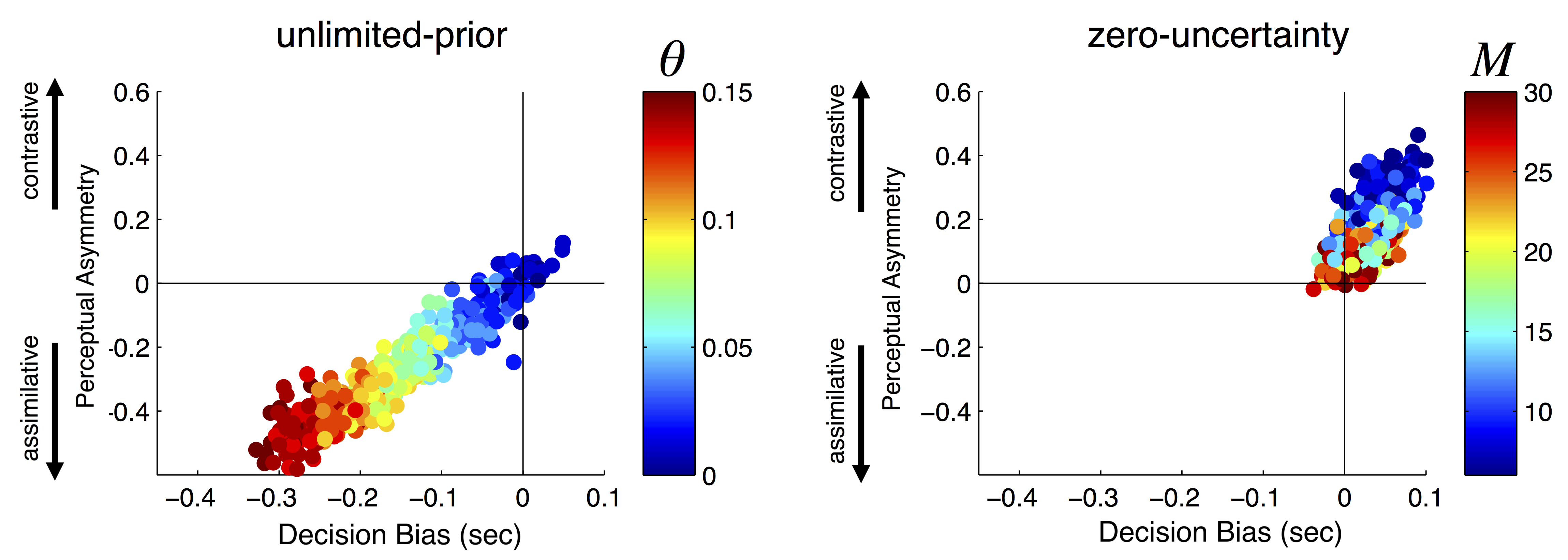

Supplement: Figure S4 — Comparison of model parameters between two alternative models. In the unlimited-prior and zero-uncertainty models, only one parameter, θ or M, was varied. Colored points display the value of the varied parameter. In the unlimited-prior model, lower values of θ (in seconds) are associated with less carryover, whereas in the zero-uncertainty model, higher values if M (in trials) lead to less carryover. (TIFF) [file pone.0100803.s004.tiff]

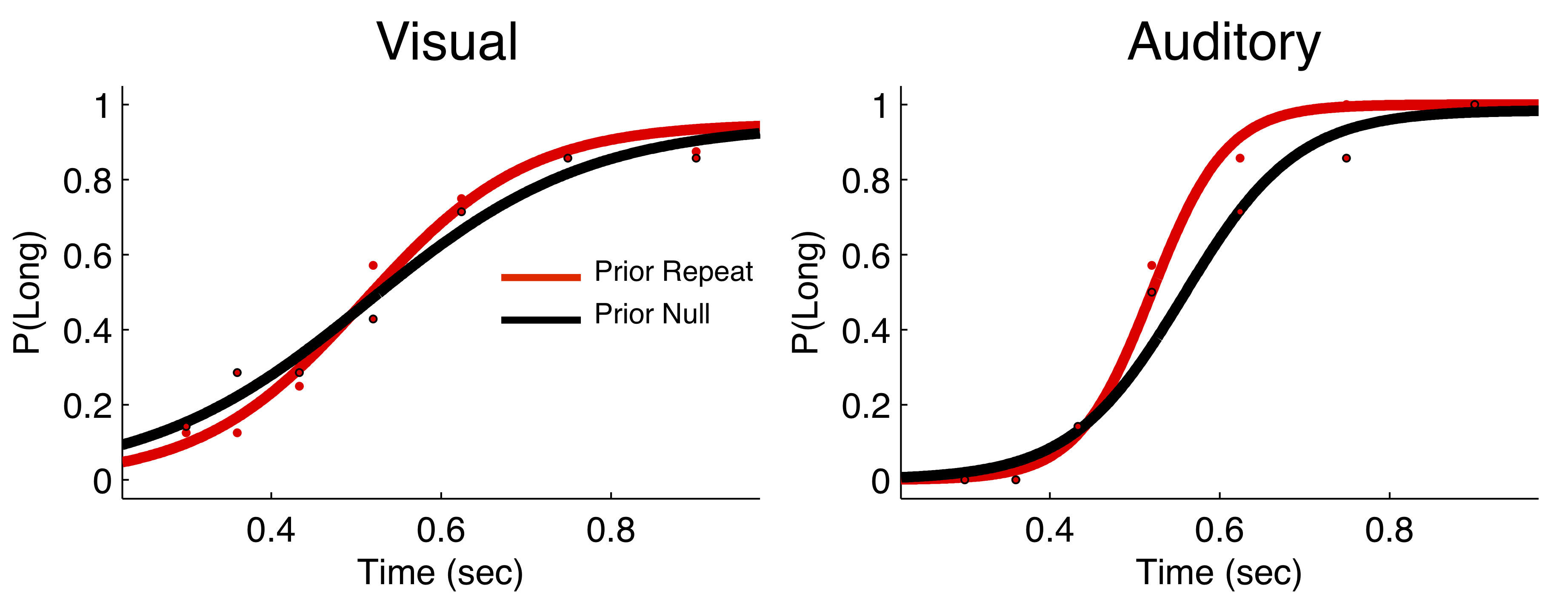

Supplement: Figure S5 — Repetition effects. For visual and auditory participants, grand-averaged psychometric curves are displayed representing the conditions where the same interval was presented twice (Prior Repeat), or the prior interval was a Null event, where participants viewed a blank screen. No differences in the bisection point were observed for either condition in either modality. (TIFF) [file pone.0100803.s005.tiff]

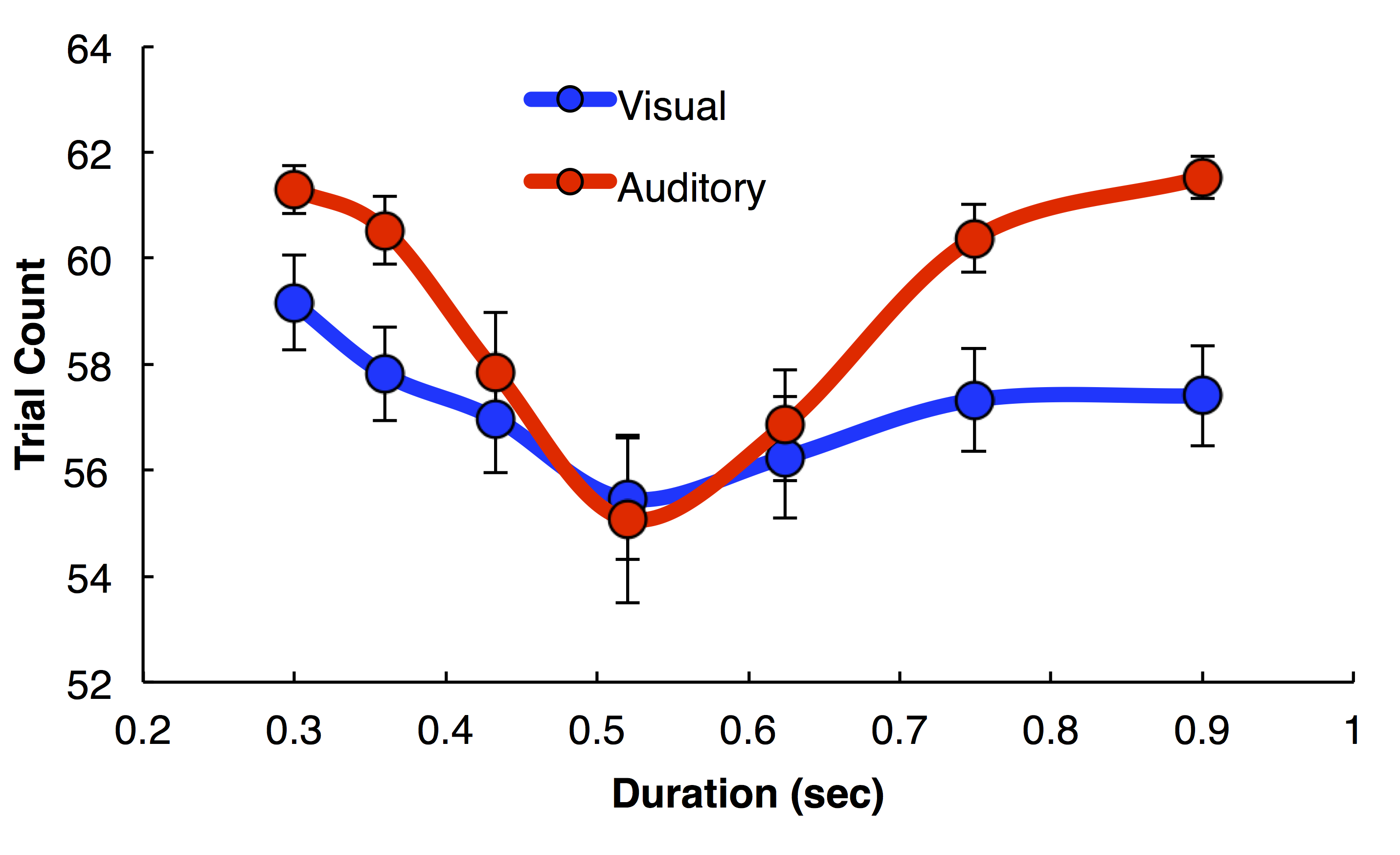

Supplement: Figure S6 — Number of trials removed after RT cutoff of 1000 ms for each duration (max = 64). Significantly more trials were removed for visual than auditory stimuli [F(6,384) = 2.930, p = 0.008]. Both modalities demonstrated an effect of duration [F(6,384) = 15.688, p<0.001], with more trials being removed around the middle range of durations. (TIFF) [file pone.0100803.s006.tiff]

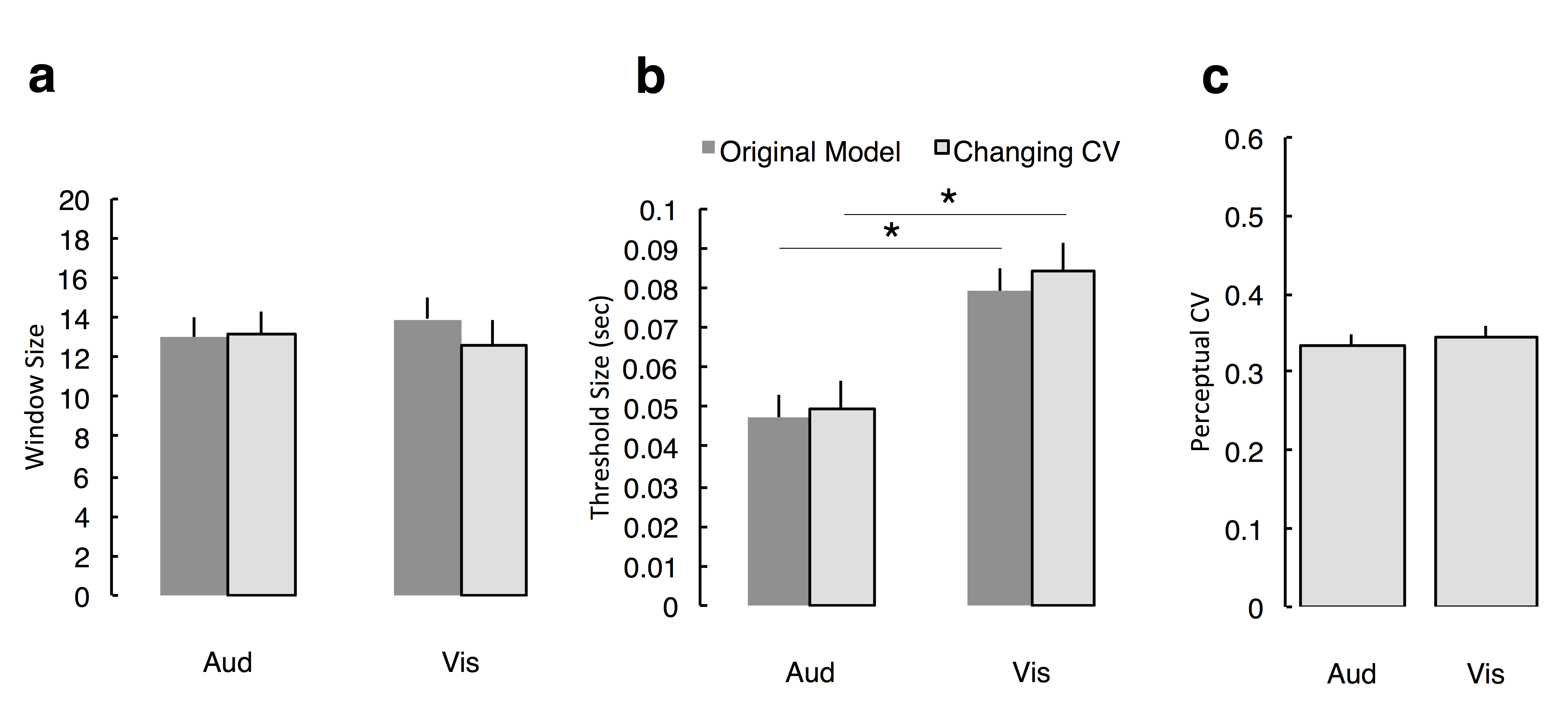

Supplement: Figure S7 — Fits to individual participant data of the original model with perceptual variability as a free parameter. Model fits are shown next to the corresponding fit values from the original model from Figure 6. The model with variability as a free parameter (“Changing CV”) produced values of window size (a) and threshold size (b) that were not significantly different from their counterparts in the original model (all p>0.05), and also produced threshold values that were significantly higher for visual than auditory participants (p<0.05). (c) Additionally, no difference between the variability of perceptual measurements was found for fits to auditory and visual participants (p>0.05). (TIFF) [file pone.0100803.s007.tiff]

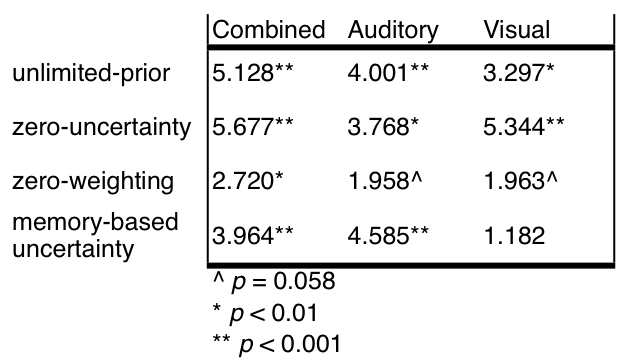

Supplement: Table S1 — Paired t-test values, displayed as t -statistics, comparing the original model fits against each of the alternative model fits. Separate comparisons are displayed for the entire set of subjects, and separated between auditory and visual subjects. (TIFF) [file pone.0100803.s008.tiff]
